# 5‐FU inhibits migration and invasion of CRC cells through PI3K/AKT pathway regulated by MARCH1

**DOI:** 10.1002/cbin.11493

**Published:** 2020-10-29

**Authors:** Nuan Wang, Lijuan Yang, Juanjuan Dai, Yan Wu, Ranran Zhang, Xingfang Jia, Chengxia Liu

**Affiliations:** ^1^ Department of Gastroenterology Binzhou Medical University Hospital Binzhou China; ^2^ Cancer Research Laboratory Binzhou Medical University Hospital Binzhou China

**Keywords:** 5‐FU, colorectal cancer, EMT, invasion, MARCH1, migration

## Abstract

Colorectal cancer is a major health problem with a significant impact on the patients' quality of life. 5‐Fluorouracil is the most common chemotherapy drug used for this type of cancer. While its molecular mechanism is the inhibition of DNA synthesis via the inhibition of thymine nucleotide synthetase, its complete anticancer mechanism is not clear. Membrane‐associated RING‐CH‐1 (MARCH1) is an E3 ubiquitin ligase that plays an important role in antigen presentation. However, MARCH1 has not been studied in the context of colorectal cancer. In this study, we demonstrated that MARCH1 is highly expressed in colorectal cancer tissues and cell lines. Furthermore, migration and invasion of colorectal tumor cells were inhibited via transfection with small interfering RNAs to suppress the expression of MARCH1. The western blot analysis showed that MARCH1 regulates epithelial–mesenchymal transition and the PI3K/AKT pathway. Moreover, 5‐fluorouracil inhibited the proliferation, migration, and invasion of tumor cells, via the targeting of MARCH1 and the consequent downregulation of the PI3K/AKT pathway, impacting the progression of epithelial–mesenchymal transition. In conclusion, our study shows that MARCH1 may play a role as an oncogene in colorectal cancer and may represent a new target molecule of 5‐fluorouracil.

**Abbreviations**
APCs: antigen‐presenting cells
CRC: colorectal cancer
DCs: dendritic cells
EMT: epithelial–mesenchymal transition
FU: 5‐fluorouracil
MARCH1: membrane‐associated RING‐CH‐1

## 1 INTRODUCTION

Colorectal cancer (CRC) is one of the most common tumors in the digestive tract. The pathogenesis of CRC is unclear; it may be related to the malignant change of colorectal polyps, chronic inflammation of the colorectal mucosa, dietary habits, or heredity factors (Mármol et al., 2017; Thanikachalam & Khan, 2019). Despite considerable improvements in the treatment of CRC, its incidence is increasing due to metastasized tumors (Piawah & Venook, 2019). Of note, metastasis and invasion are also the leading causes of poor tumor prognosis. Epithelial–mesenchymal transition (EMT) plays an important role in tumor migration and invasion. It refers to the process of the conversion of epithelial cell morphology into an interstitial phenotype under the stimulation of external signals (Cao et al., 2015). Therefore, studying EMT and its role in invasion and proliferation in the context of CRC cells may provide new therapeutic targets to prevent metastasis and recurrence.

5‐Fluorouracil (5‐FU) is one of the most used and effective chemotherapy drugs for CRC (Grem, 2001). Most patients receive 5‐FU‐based treatment regimens; both intravenous and oral 5‐FU for CRC have become the main systemic treatment since the 1990s. 5‐FU is an antimetabolite drug; the hydrogen at position C‐5 of uracil is replaced by fluorine. Many studies have focused on the bio‐modulation of 5‐FU to improve its therapeutic anticancer effectiveness and selective cytotoxicity (Goirand et al., 2018). Owing to genetic and epigenetic differences of CRC patients, however, the incidence of 5‐FU resistance is gradually increasing. Consequently, exploring specific targeted molecules or pathways of 5‐FU resistance may help improve patients' outcomes in the future.

The membrane‐associated RING‐CH (MARCH) family comprises E3 ubiquitin ligases located on the plasma and organelle membranes, consisting of an N‐terminal ring finger and zero, two, or more C‐terminal transmembrane domains (Bauer et al., 2017). Of the 11 members of the MARCH family, some play important roles in immune regulation, such as endoplasmic reticulum degradation, protein‐energy control, and membrane transport (Lin et al., 2019; Nakamura, 2011). MARCH1, expressed by antigen‐presenting cells (APCs), dendritic cells (DCs), and B cells in lymphoid tissues (e.g., the spleen), participates in the regulation of antigen presentation via the ubiquitination of major histocompatibility complex (MHC)‐II and CD86 (Corcoran et al., 2011; Wilson et al., 2018). Many studies have reported the role of MARCH1 in the immune system. However, MARCH1 also takes part in the development of cancers. A study found that MARCH1 is overexpressed in ovarian cancer tissues and that MARCH1 silencing inhibited the proliferation, invasion, and migration of SKOV3 cells via the mediation of the nuclear factor‐κB (NF‐κB) and the Wnt/β‐catenin pathways (Meng et al., 2016). However, there is no research on the role of MARCH1 in CRC.

In this study, we aimed to investigate the role and expression of MARCH1 in CRC tissues and cell lines. Furthermore, we also investigated its role in metastasis and tumor invasion, as well as its response to 5‐FU treatment. We demonstrate that MARCH1 is highly expressed in CRC tumor tissues and cell lines. Moreover, the results show that MARCH1 downregulation inhibits EMT and regulates the migration and invasion of CRC cells via the PI3K/AKT pathway. Furthermore, we show that the levels of MARCH1 are decreased in CRC cells after treatment with 5‐FU. Notably, we identified the underlying mechanism: 5‐FU not only downregulates the expression of MARCH1, but also inhibits the expression of the PI3K/AKT pathway to repress the proliferation, migration, and invasion of CRC in vitro. This article mainly proposes that 5‐FU plays a role in the inhibition of CRC malignant biological behaviors via the downregulating of MARCH1 through the PI3K/AKT pathway.

## 2 MATERIALS AND METHODS

### 2.1 CRC clinical samples

Twenty clinical CRC samples and adjacent peritumoral tissues were collected from patients who had experienced radical resection of CRC at Binzhou Medical University Hospital. Tumor tissues and adjacent tissues were collected within 30 min from the body. The adjacent tissues were taken from noncancerous tissues about 5 cm away from the tumor tissue. After taking the tissues, they were divided into about 0.5 cm tissue pieces and stored in a liquid nitrogen tank. These patients did not undergo radiotherapy and chemotherapy and signed informed consent before surgery. This study was approved by the Ethics Committee of Binzhou Medical University Hospital.

### 2.2 Cell culture, small interfering RNA (siRNA) transfection, and chemicals

All the cells were originally owned by the laboratory. The intestinal epithelial cell NCM460 cultured in McCoy's 5 A medium (Biological Industries) and the human CRC cell lines SW480 and DLD‐1 cultured in Dulbecco's modified Eagle's medium (DMEM; Biological Industries) and RPMI‐1640 medium (Biological Industries), respectively supplemented with 10% fetal bovine serum (FBS; Biological Industries) and 1% penicillin–streptomycin solution (Hyclone) at 37℃ in 5% CO_2_ atmosphere, and 0.25% trypsin (Biological Industries) was used to passage the cell lines till they reached 90% confluence. The cells which were planted (3 × 10^5^ per well) on six‐well culture dishes or planted (5 × 10^3^ per well) on 96‐well culture dishes were transfected with 50 nM siRNA‐MARCH1 using 5 µl Lipofectamine 2000 (Invitrogen) according to the manufacturer's protocol. Two siRNAs against MARCH1 and negative control (NC) siRNAs were purchased by Genepharma. The sequences for the MARCH1 siRNA were: siMARCH1‐1, 5′‐CAGGAGGUCUUGUCUUCAUTT‐3′ and 5′‐AUGAAGACAAGACCUCCUGTT‐3′ siMARCH1‐2, 5′‐GGUAGUGCCUGUACCACAATT‐3′ and 5′‐UUGUGGUACAGGCACUACCTT‐3′ The NC siRNA sequences were: 5′‐UUCUCCGAACGUGUCACGUTT‐3′ and 5′‐ACGUGACACGUUCGGAGAATT‐3′ 5‐FU was purchased from Sigma. 5‐FU was dissolved in phosphate‐buffered saline (PBS) and was stored at −20℃.

### 2.3 RNA isolation and quantitative reverse‐transcription polymerase chain reaction (qRT‐PCR)

Total RNA was extracted using TRIzol reagent (Sangon Biotech) from cells and tumor tissues. Complementary DNA synthesis was performed with oligo (dT) by using a RevertAid First Synthesis Kit (Thermo Fisher Scientific) according to the manufacturer's instructions. AceQ® qPCR SYBR® Green Master Mix (Vazyme) was used for transcript quantification with specific primers. Expression levels were quantified using the method with glyceraldehyde‐3‐phosphate dehydrogenase (GAPDH) as control. Primers were designed and synthesized by Sangon Biotech. The sequences of the primers used were as followed. MARCH1: the forward sequence was 5′‐CACGTTCCACGTCATCGCCGT‐3′, the reverse sequence was 5′‐ ATGGCCATTCCAGCACACCTTGC‐3′. GAPDH: the forward sequence was 5′‐CTCCTCCTGTTCGACAGTCAGC‐3′, the reverse sequence was 5′‐CCCAATACGACCAAATCCGTT‐3′.

### 2.4 Western blot analysis

We added liquid nitrogen to 20–50 mg tissue, grinded it into powder, and then added an appropriate amount of protein lysis buffer (Beyotime). Whole‐cell protein lysates were extracted using protein lysis buffer, and the protein concentrations of tissue and cell were determined by the BCA assay (Solarbio). The lysates were boiled in sodium dodecyl sulfate polyacrylamide gel electrophoresis (SDS‐PAGE) sample loading buffer (EpiZyme) for 5–10 min at 99°C and separated on SDS‐PAGE and transferred to polyvinylidene difluoride membranes (Millipore) by electroblotting, and after blocking in 5% nonfat milk (Sangon Biotech) for 2–3 h. Then the membrane was incubated with primary antibodies against anti‐MARCH1 (Immunoway), anti‐AKT (ImmunoWay), anti‐PI3K (Immunoway), anti‐pAKT (Immunoway), anti‐pPI3K (Immunoway), anti‐E‐cadherin (Cell Signaling Technology), anti‐Vimentin (Proteintech), anti‐β‐actin (Proteintech), anti‐tubulin‐α (Cell Signaling Technology) at 4°C overnight and then left with the secondary antibodies peroxidase‐conjugated goat anti‐rabbit (BOSTER) or peroxidase‐conjugated goat anti‐mouse (BOSTER) for 30 min at 37°C. Finally, the membranes were quantified using an enhanced chemiluminescence signal (ECL, EpiZyme). Photometric analyses of immunoblots were carried out using the Image Lab software package. The quantitative analysis through ImageJ software.

### 2.5 Cell proliferation assay

Cell proliferation assays were performed using a cell counting kit‐8 (CCK‐8; Dojindo). According to the manufacturer's instructions, 2000 cells were seeded in 96‐well plates overnight before treatment. CCK‐8 reagent was added to each well, and after incubation with the reagent for 2 h at 37°C. The absorption and reference wavelength was measured at 450 nm. After normalizing the 0 h optical density average value, Graphpad Prism 7.0 software was used to draw the proliferation curve of each group of cells over time.

### 2.6 Colony formation assay

One thousand cells were seeded in six‐well plates per well, treated with 5‐FU after 24 h, and grown for 14 days. Then the colonies were stained with 0.1% crystal violet solution (Sangon Biotech), washed with PBS, and imaged with a photomicroscope. Colonies containing over 50 cells were counted.

### 2.7 Cell apoptosis assay

The apoptosis assays were performed using an Annexin V, Fluorescein Isothiocyanate (FITC) Apoptosis Detection Kit (Dojindo) according to the manufacturer's instructions. We collected at least 10,000 cells after transfection, washed them twice with cold PBS, and used 100 µl annexin V binding solution to make cell suspension. The cell suspension was incubated with 5 μl of annexin V‐FITC and 5 μl of propidium iodide (PI) for 15 min, followed by apoptosis analysis by flow cytometry (BD Biosciences).

### 2.8 Wound healing assay

SW480 and DLD‐1 cells were seeded in six‐well plates at a density of 1 × 10^6^ and cultured in DMEM or 1640 containing 10% FBS to 90% confluence. The cells were scraped with a 200‐µl pipette tip to create wounds and then supplemented with a serum‐free medium to inhibit proliferation. Images of the wounds were captured after 0, 24, and 48 h using a photomicroscope. The quantitative analysis through ImageJ software. The migratory ability of the cells was calculated as the ratio of the open area after 24 and 48 h to the open area at 0 h.

### 2.9 Transwell assay

Cell migration and invasion assay were performed using 6.5 mm transwell insert chambers with an 8.0 µm pore polycarbonate membrane. The cells (3000) were cultured in serum‐free medium, placed in the upper chambers, and coated with Matrigel basement membrane matrix (Corning) for 2 h at 37°C before the cells were added. The medium with 20% FBS was added to the down chamber. The cells were incubated for 24–48 h, and cells that did not migrate through the pores were removed with a cotton swab. Then the upper chambers were fixed in 4% paraformaldehyde, stained with 0.5% crystal violet (Sangon Biotech), and counted under a photomicroscope. However, there was no need for the Matrigel coating for the cell migration assay.

### 2.10 Immunohistochemistry

All tissue microarray slide containing tumor and adjacent tissues were provided by Binzhou Medical University Hospital. All tissues were fixed in 4% paraformaldehyde overnight and subsequently embedded in paraffin wax. The embedded‐tissues were cut into 4‐μm sections which were stained for analysis. The sections were deparaffinized using dimethylbenzene followed by antigen retrieval by heating for 20 min in ethylenediaminetetraacetic acid buffer (pH = 9.0) in a microwave oven. Then using 3% hydrogen peroxide to block endogenous peroxidase and tissues were incubated with primary antibodies or PBS as a negative control at 1:300 dilutions with 5% bovine serum albumin (BSA) at 4°C overnight and then left with secondary antibodies diluted at 1:200 by 5% BSA at 37°C for 30 min. Lastly, using Diaminobenzidine Detection System to develop color reaction according to the instruction. Immunostaining analysis was performed by experienced pathologists. Five fields were randomly taken from each section to assess the staining intensity of tumor and nontumor fields. Average positivity from each sample was calculated as the mean positivity from all fields. The staining intensity score of tumor tissues and adjacent nontumor tissues was as follows: 0 (negatively stained), 1 (weakly stained), 2 (moderately stained), 3 (strongly stained).

### 2.11 Statistic analysis

SPSS 23.0 was used to analyze the data, and GraphPad Prism 7.0 software was used for statistical drawing. The measurement data is expressed as mean ± standard deviation (mean ± *SD*). The *t* test or analysis of variance is used to compare the measurement data that follow the normal distribution. The nonparametric rank‐sum test is used when the normal distribution is not followed. Count data is analyzed by *χ* ^2^ test. Correlation between MARCH1 protein expression and clinicopathological characteristics using Spearman

Correlation analysis. *p < .05, **p < .01, and ***p < .001 were considered statistically significant.

## 3 RESULTS

### 3.1 MARCH1 is highly expressed in CRC tissues and cell lines

**Figure 1:**
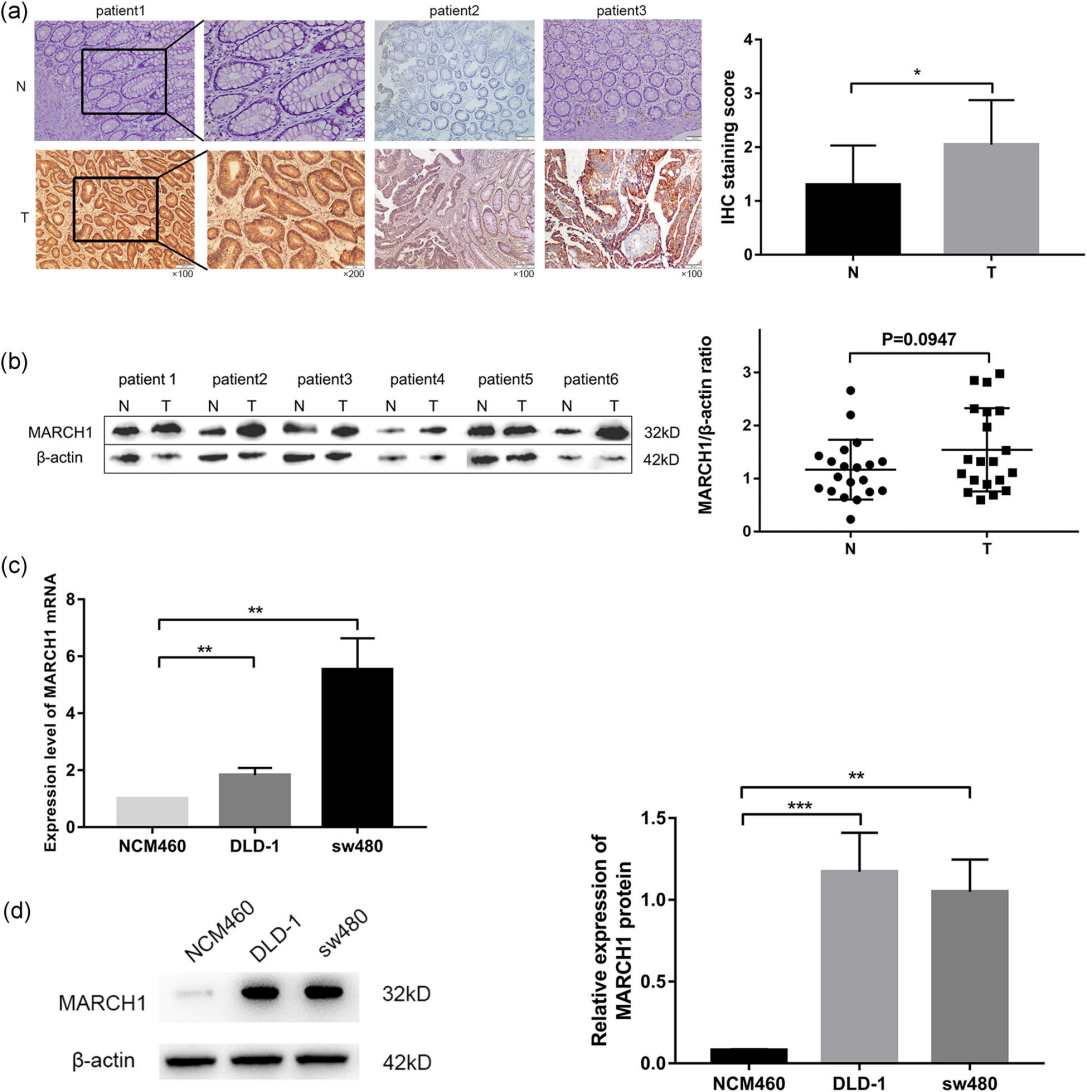
Expression level of MARCH1 in CRC tissues and cell lines. (a) Immunohistochemistry (IHC) analysis of the expression level of MARCH1 in CRC tumor tissues (T) and adjacent nontumor tissues (N); IHC staining score of MARCH1 in 20 CRC patients. (b) Western blot analysis assay of the expression level of MARCH1 in CRC tumor tissues (T) and adjacent nontumor tissues (N), β‐actin was regarded as a control gene. (c, d) qRT‐PCR and western blot analysis assay detected the expression level of MARCH1 mRNA and protein in epithelial cell line and CRC cell line. Results were presented as mean ± *SD* of three independent experiments. **p < .01, ***p < .001. CRC, colorectal cancer; MARCH1, membrane‐associated RING‐CH‐1; mRNA, messenger RNA; qRT‐PCR, quantitative reverse‐transcription polymerase chain reaction; *SD*, standard deviation

We used immunohistochemistry and western blot analysis assays to detect the expression levels of MARCH1 in human CRC tissues (Figure 1a,b). We collected 20 primary CRC samples and the results showed that 12 (60%) samples showed high expression levels of MARCH1 (tumor tissues vs. adjacent nontumor tissues). Then we divided the 20 CRC patients into MARCH1 high and low expression groups according to the expression levels of MARCH1 (as per the mean ± *SD* values) in CRC tissues as per western blot analysis results and analyzed the relationship between the expression of MARCH1 and clinicopathological characteristics (Table 1). No association was found, probably due to the small sample size. Furthermore, the messenger RNA (RNA) and protein levels of MARCH1 in intestinal epithelial cells (NCM460) and CRC cell lines (DLD‐1 and sw480) were quantified using qRT‐PCR and western blot analysis, respectively. The results showed that sw480 and DLD‐1 cells expressed higher levels of MARCH1 than NCM460 cells, at both the mRNA (Figure 1c) and protein (Figure 1d) levels.

**Table 1:** Correlation of MARCH1 expression with clinicopathological characteristics in 20 CRC patients

	Patients	Expression of MARCH1			
	(*n *= 20)	Low	High	r	p
Gender					
Male	12	5	7	.042	.862
Female	8	3	5		
Age (years)					
<60	3	2	1	.229	.332
≥60	17	6	11		
Tumor diameter (cm)					
<4	7	2	5	−.171	.471
≥4	13	7	7		
Tumor site					
Colon	8	3	5	−.042	.862
Rectum	12	5	7		
Differentiation					
Low	1	0	1	.187	.429
Medium‐high	19	8	11		
Infiltration depth					
Mucosa and submucosa	2	0	2	−.047	.845
Muscularis propria	3	2	1		
Adventitia	15	6	9		
Lymph node metastasis					
Negative	17	7	10	.057	.811
Positive	3	1	2		
T stage					
T1–T2	5	2	3	.000	1.000
T3–T4	15	6	9		
N stage					
N0	17	7	10	.057	.811
N1–N2	3	1	2		
M stage					
M0	19	8	11	0.187	.429
M1	1	1	0		
AJCC stage					
I–II	18	7	11	−.068	.776
III–IV	2	1	1		

### 3.2 MARCH1 knockdown does not impact CRC cell proliferation and apoptosis

MARCH1 was knocked down using siRNAs in DLD‐1 and sw480 cells. The knockdown efficiency after transfection with siMARCH1‐1 and siMARCH1‐2 versus the negative control (siNC) was evaluated (Figure 2a). Both siMARCH1‐1 and siMARCH1‐2 could effectively knockdown the expression of MARCH1. Microscopy images of DLD‐1 and sw480 cells 48 h after transfection were also acquired (Figure 2b). There was no significant difference in cell number between siMARCH1 groups and siNC group. To further investigate whether MARCH1 influenced CRC cells’ proliferation, we used the CCK‐8 assay to determine cell growth after transfection with siRNAs. The viability of CRC cell lines transfected with siRNAs was not significantly different compared with that of the siNC group (Figure 2c,d). These results indicate that the high MARCH1 expression in CRC cell lines does not influence their proliferation. Moreover, to explore the effect of MARCH1 knockdown on cell apoptosis, we used flow cytometry (Figure 2e). The results showed that the downregulation of MARCH1 has no significant effect on cell apoptosis.

Figure 2Downregulation of MARCH1 can not inhibit proliferation and apoptosis in CRC cell lines. (a) Western blot analysis assay showed that the expression level of MARCH1 decreased after using two siRNAs for 48 h compared with siNC in CRC cell lines. (b) Microscope images of the CRC cells of MARCH1 siRNA interference for 48 h using ×100 image lens (scale bar: 200 µm). (c) The cell viability of the DLD‐1 and sw480 cells transfected with MARCH1 siRNAs and siNC at 48 h using CCK‐8 assay. (d) The cell growth curve of the DLD‐1 and sw480 cells transfected with MARCH1 siRNAs and siNC at 0, 24, 48, and 72 h using CCK‐8 assay.(e) The cell apoptosis rate of the DLD‐1 and sw480 cells transfected with MARCH1 siRNAs and siNC at 48 h. Results were presented as mean ± *SD* of three independent experiments. CCK‐8, cell counting kit‐8; CRC, colorectal cancer; MARCH1, membrane‐associated RING‐CH‐1; *SD*, standard deviation; siRNA, small interfering RNA

**Figure:**
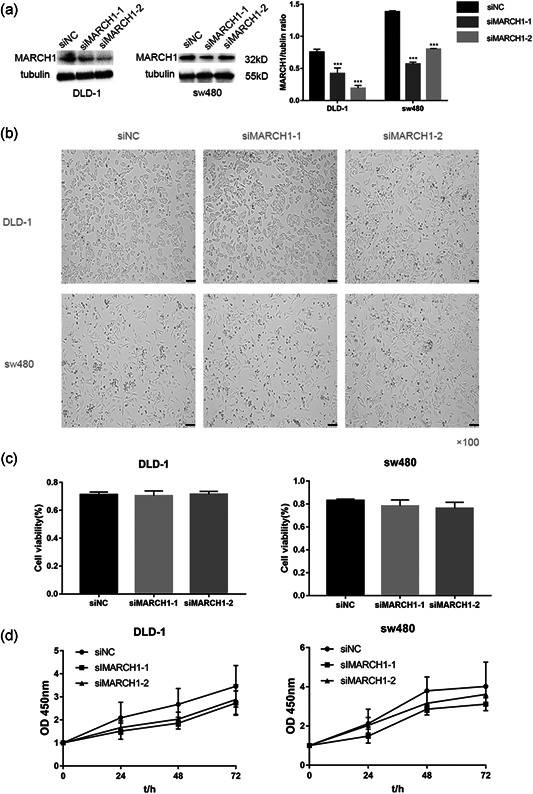

**Figure:**
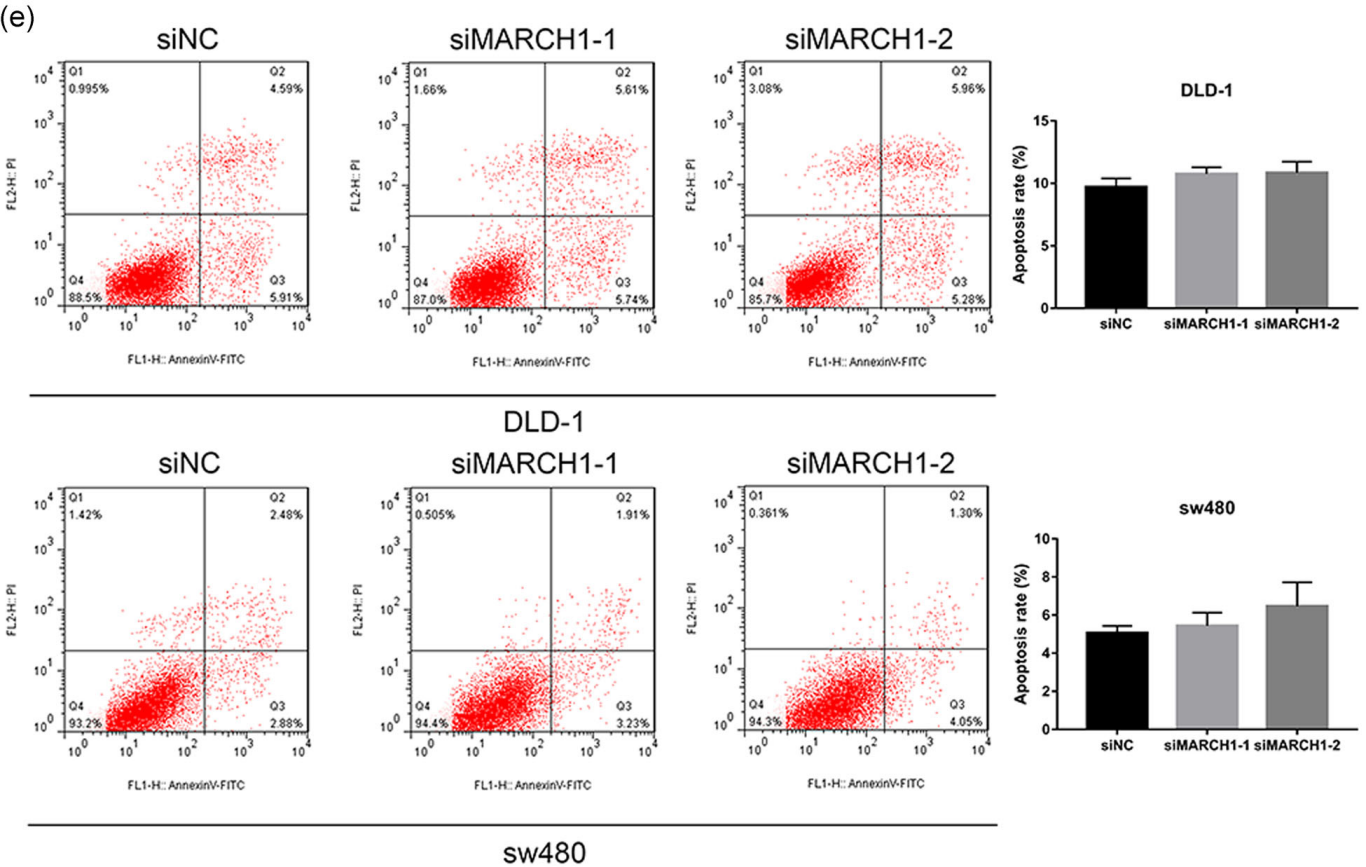

### 3.3 MARCH1 knockdown suppresses CRC cell migration, invasion, and EMT via the PI3K/AKT pathway

Wound healing and transwell assays were performed to determine the cell migration and invasion ability of CRC cell lines, respectively. Interestingly, the scratch experiment showed that the downregulation of MARCH1 led to decreased cell migration (Figure 3a). Moreover, the transwell invasion assay revealed that MARCH1 has an effect on the invasion ability of CRC cells (Figure 3b). EMT is the most common phenomenon behind tumor metastasis and invasion. Interestingly, we found that E‐cadherin increased and Vimentin decreased after transfection of siRNAs targeting MARCH1 (Figure 3c). To further explore the molecular mechanism linking MARCH1 to malignant biological behaviors, we used western blot analysis to investigate the PI3K/AKT pathway in the context of MARCH1 downregulation by siRNAs. The results indicated that the downregulation of MARCH1 led to the decreased expression of p‐PI3K and p‐AKT in DLD‐1 and sw480 cells at the protein level (Figure 3c). These results suggest that the knockdown of MARCH1 may suppress EMT, further inhibiting cell migration, and invasion via the inhibition of the PI3K/AKT pathway.

Figure 3Downregulated MARCH1 inhibited CRC cell migration, invasion, and EMT through suppressing PI3K/AKT pathway. (a) The migration ability of the DLD‐1 and sw480 cells transfected with MARCH1 siRNAs and siNC at 24 and 48 h after the scratch using ×40 image lens (scale bar: 200 µm). (b) The invasion ability of the DLD‐1 and sw480 cells transfected with MARCH1 siRNAs and siNC at 48 h using transwell assay (scale bar: 200 µm). (c) Protein expression of EMT, PI3K/AKT pathway molecules in CRC cell lines transfected with MARCH1 siRNAs and siNC at 48 h. Results were presented as mean ± *SD* of three independent experiments. ***p < .001. CRC, colorectal cancer; EMT, epithelial–mesenchymal transition; MARCH1, membrane‐associated RING‐CH‐1; *SD*, standard deviation; siRNA, small interfering RNA

**Figure:**
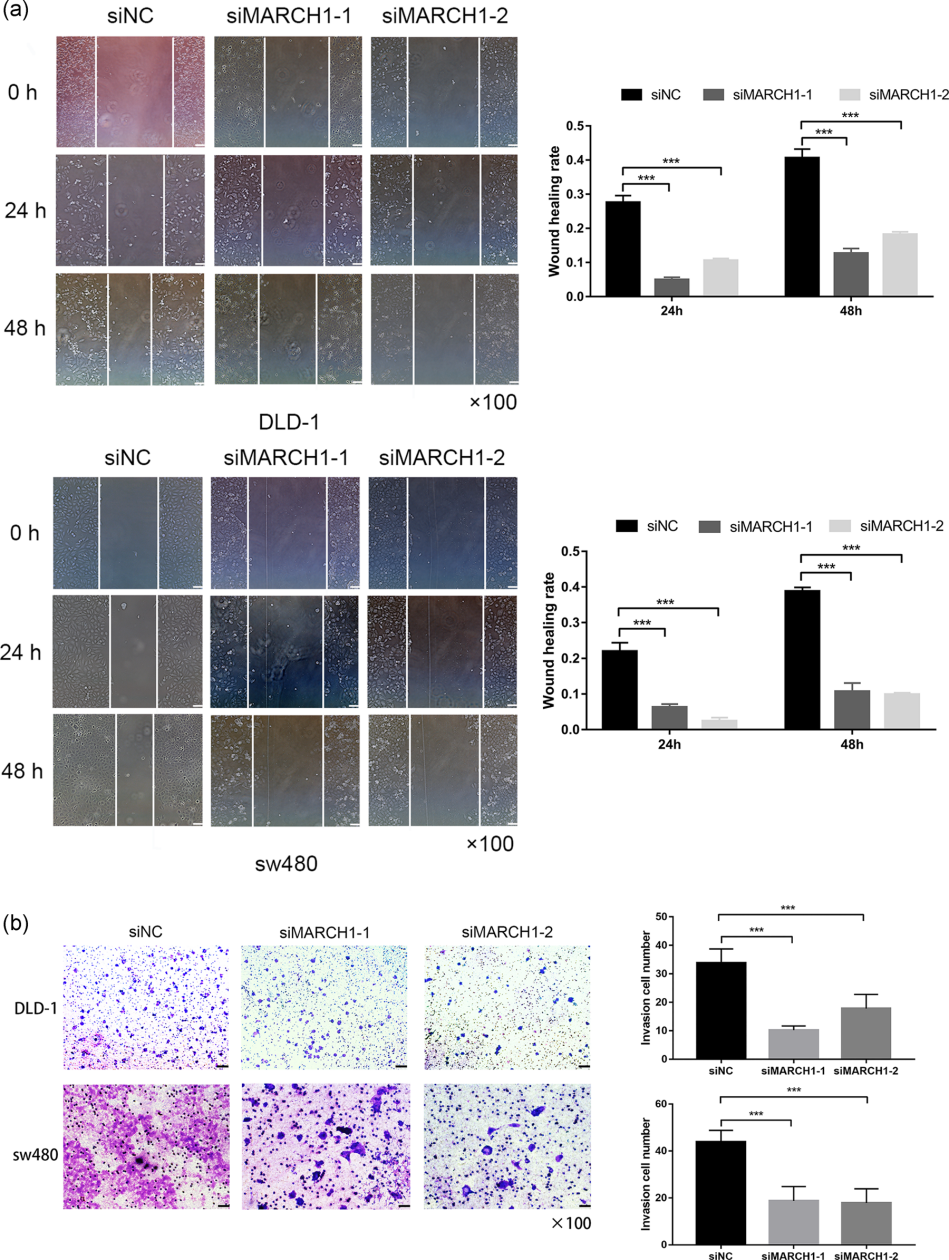

**Figure:**
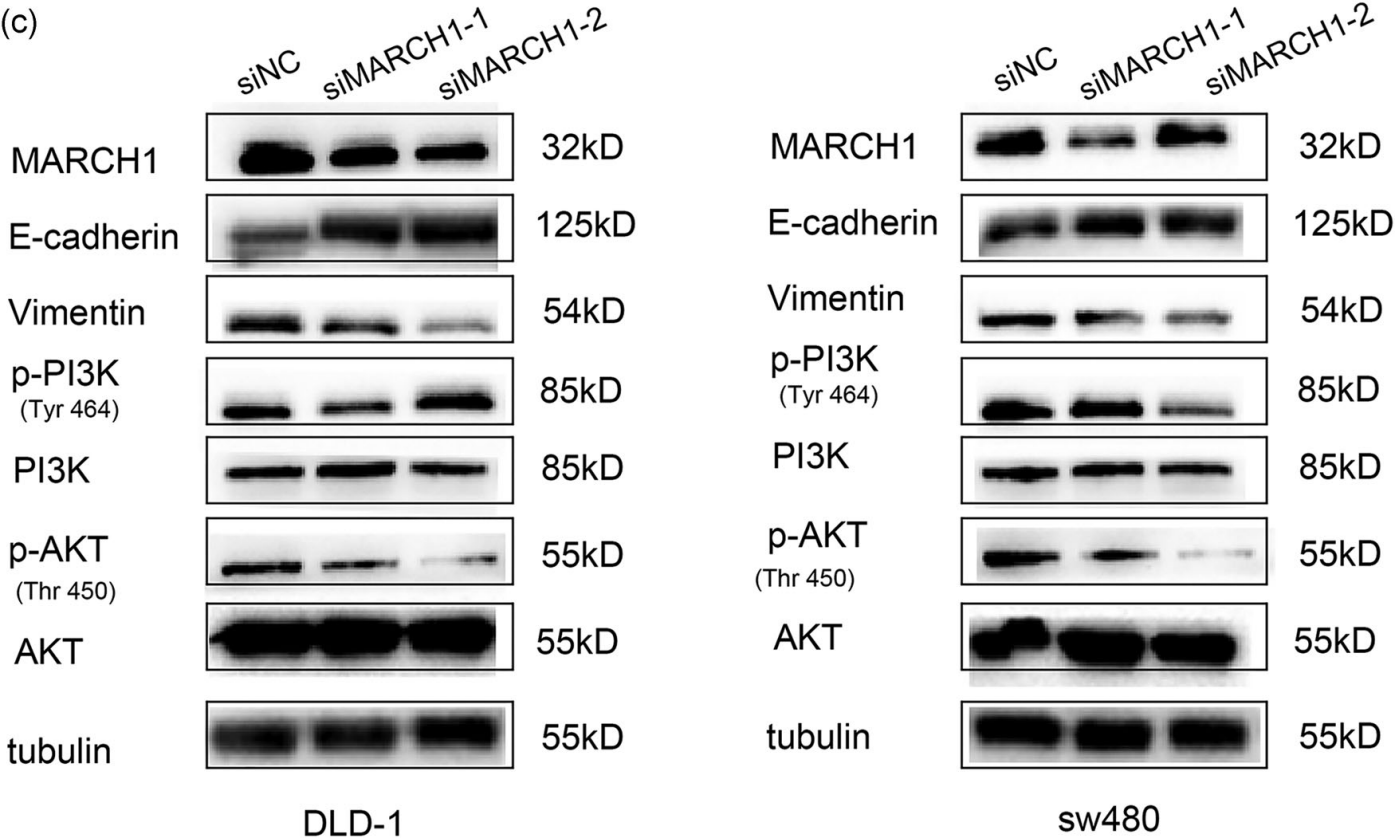

### 3.4 5‐FU inhibits CRC cell proliferation and promotes apoptosis

5‐FU is a conventional chemotherapy drug. We want to explore its new molecular mechanism to treat CRC. To understand the effects of 5‐FU on CRC cell proliferation, we treated sw480 and DLD‐1 cells with different doses of 5‐FU for 24 or 48 h (Figure 4a). Importantly, the results indicated that cell proliferation was inhibited in a dose‐dependent manner (Figure 4b). Moreover, microscopy images showed that the number of CRC cells decreased after 5‐FU treatment (Figure 4c). Then we tested the effect of 5‐FU in CRC cell lines using the colony formation assay (Figure 4d). Similarly, 5‐FU inhibited CRC cells’ cloning‐formation. Last but not least, the effect of 5‐FU on cell apoptosis was investigated via annexin V and PI staining followed by flow cytometric analysis (Figure 4e). The apoptosis rate of 5‐FU‐treated cells was significantly higher than that of the untreated group.

Figure 4Effect of 5‐FU on CRC cell proliferation and apoptosis. (a) The cell viability of the sw480 and DLD‐1 cells treated with different doses (0, 0.5, 1, 5, 10, 15, and 20 µM) 5‐FU for 48 h. (b) Microscope images of the CRC cells of treating with 0, 10, and 20 µM 5‐FU for 48 h using ×100 image lens (scale bar: 200 µm). (c) The cell viability of the sw480 and DLD‐1 cells treated with different doses (0, 10, and 20 µM) 5‐FU for 0, 24, 48, and 72 h. (d) Colony formation assay of CRC cells treated with 0, 10, and 20 µM 5‐FU for 14 days. (e) The cell apoptosis rate of the DLD‐1 and sw480 cells treated with different doses (0, 10, and 20 µM) 5‐FU for 48 h. Results were presented as mean ± *SD* of three independent experiments. **p < .01, ***p < .001. 5‐FU, 5‐fluorouracil; CRC, colorectal cancer; *SD*, standard deviation

**Figure:**
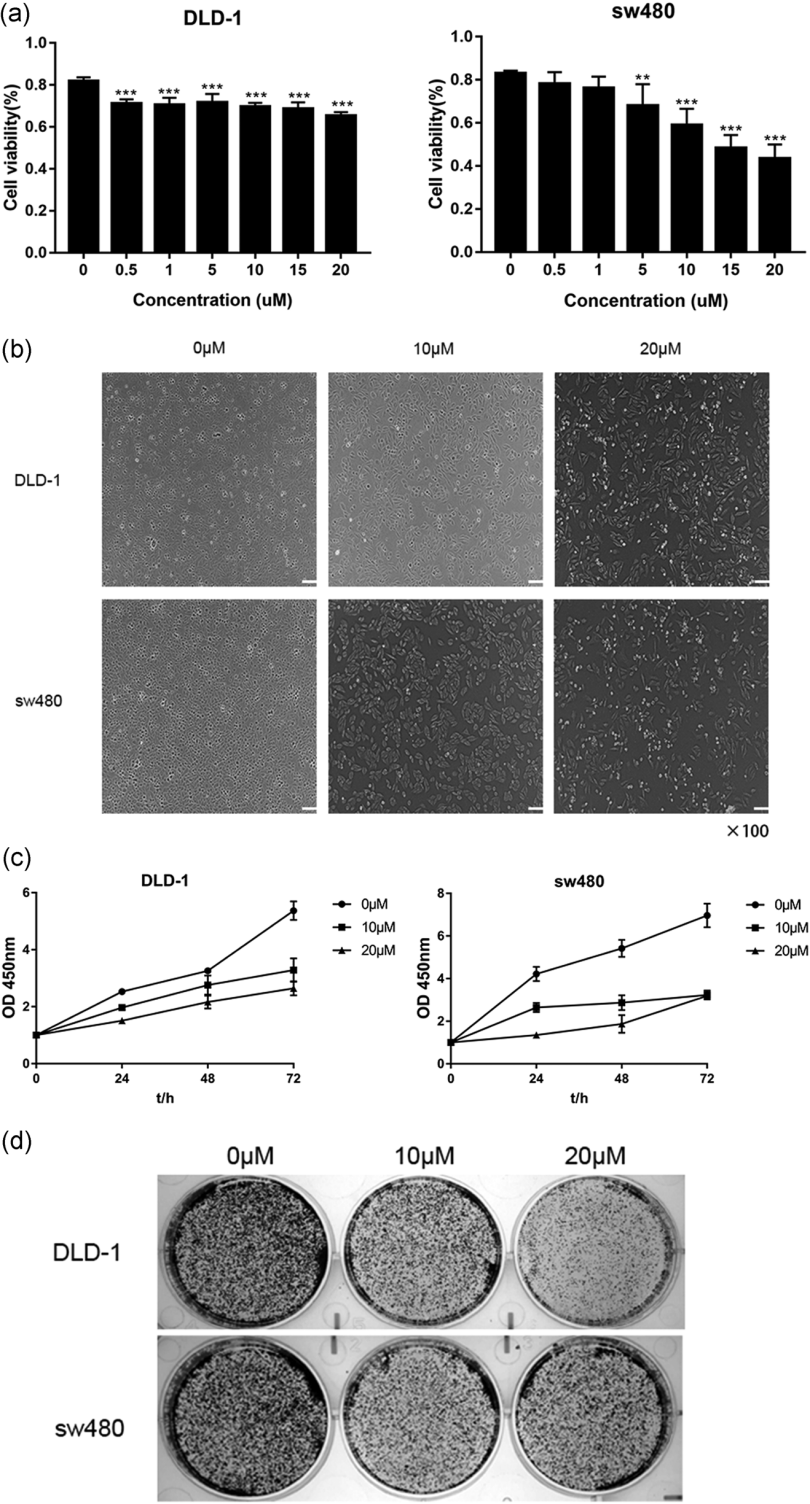

**Figure:**
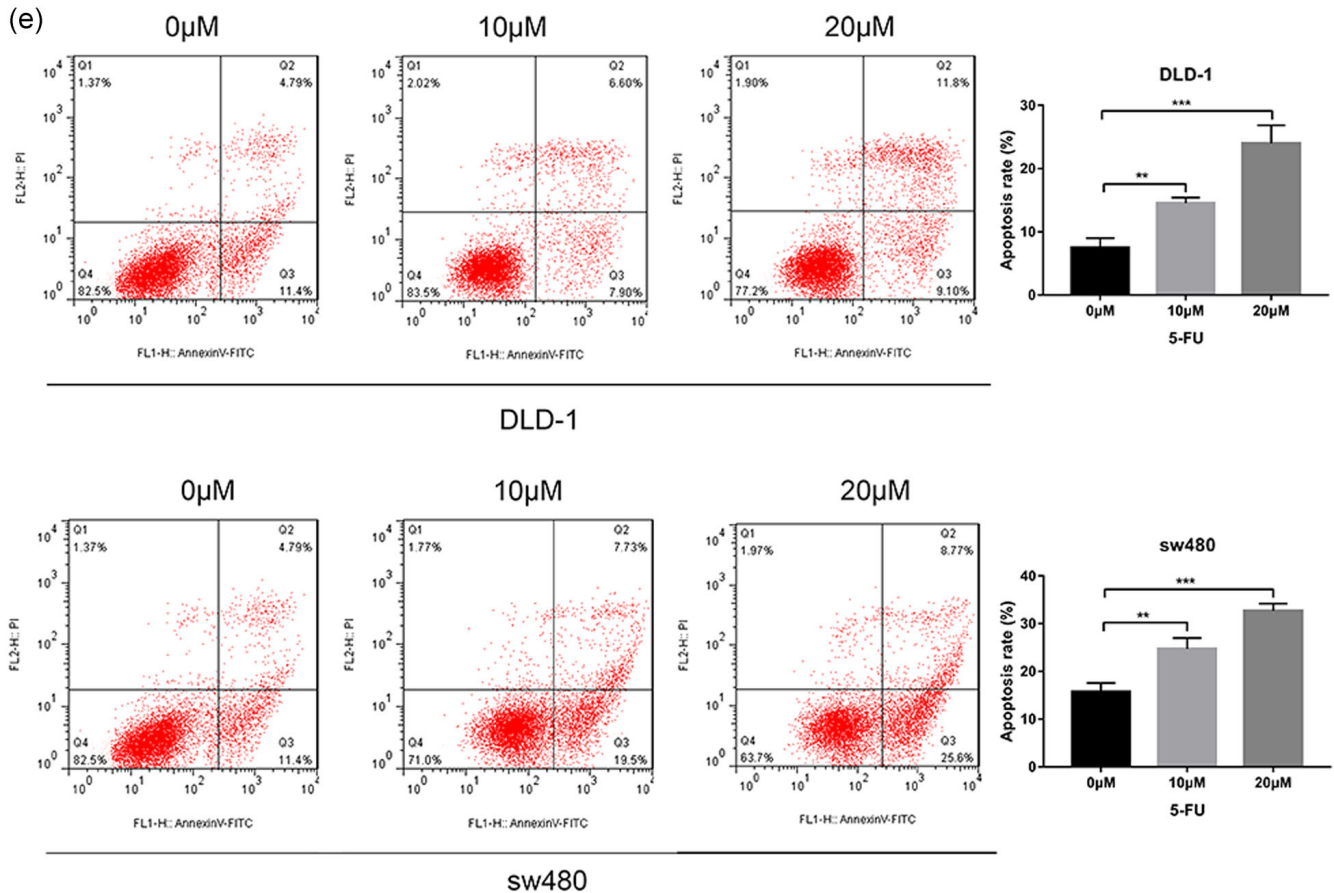

### 3.5 5‐FU inhibits cell migration, invasion, and EMT via the PI3K/AKT pathway

Compared with the blank group, 5‐FU treatment inhibited cell migration in a dose‐dependent manner (Figure 5a). Moreover, the transwell assay showed that CRC cell invasion was negatively impacted by 5‐FU treatment (Figure 5b). Interestingly, the western blot analysis showed that the protein levels of MARCH1 were dose‐dependently downregulated CRC cells after 5‐FU treatment. The results indicated that 5‐FU works through the MARCH1 pathway. To clarify the molecular mechanism of 5‐FU, we tested the expression of EMT and PI3K/AKT pathway molecules. Remarkably, the results showed that 5‐FU inhibited the expression of p‐PI3K, p‐AKT. Moreover, E‐cadherin was elevated and Vimentin was decreased after 5‐FU treatment (Figure 5c). These results suggest that 5‐FU treatment inhibits malignant behaviors of CRC cells, partially due to the regulation of the MARCH1 and PI3K/AKT pathways.

Figure 5Effect of 5‐FU on CRC cell migration, invasion, EMT, and PI3K/AKT pathway. (a) Wound healing assay demonstrated that the migration ability in CRC cells after 24 and 48 h of 5‐FU treatment (0, 10, and 20 µM) using ×40 image lens (scale bar: 200 µm). (b) Transwell assay demonstrated that the invasion ability in CRC cells after 48 h of 5‐FU treatment (0, 10, and 20 µM) (scale bar: 200 µm). (c) Western blot analysis assay showed the protein expression of MARCH1, EMT, PI3K/AKT pathway molecules in CRC cells after 48 h of 5‐FU treatment (0, 10, and 20 µM). Results were presented as mean ± *SD* of three independent experiments. *p < .05, **p < .01, ***p < .001. 5‐FU, 5‐fluorouracil; CRC, colorectal cancer; EMT, epithelial–mesenchymal transition; *SD*, standard deviation

**Figure:**
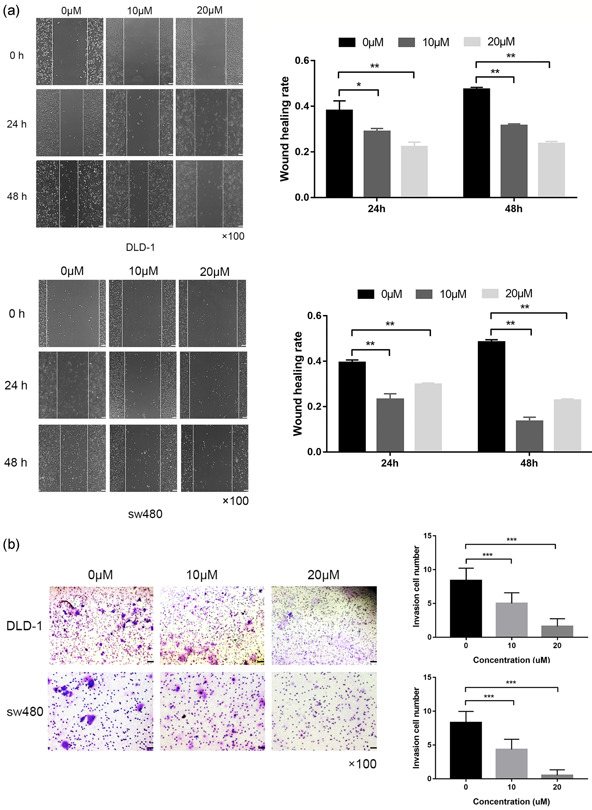

**Figure:**
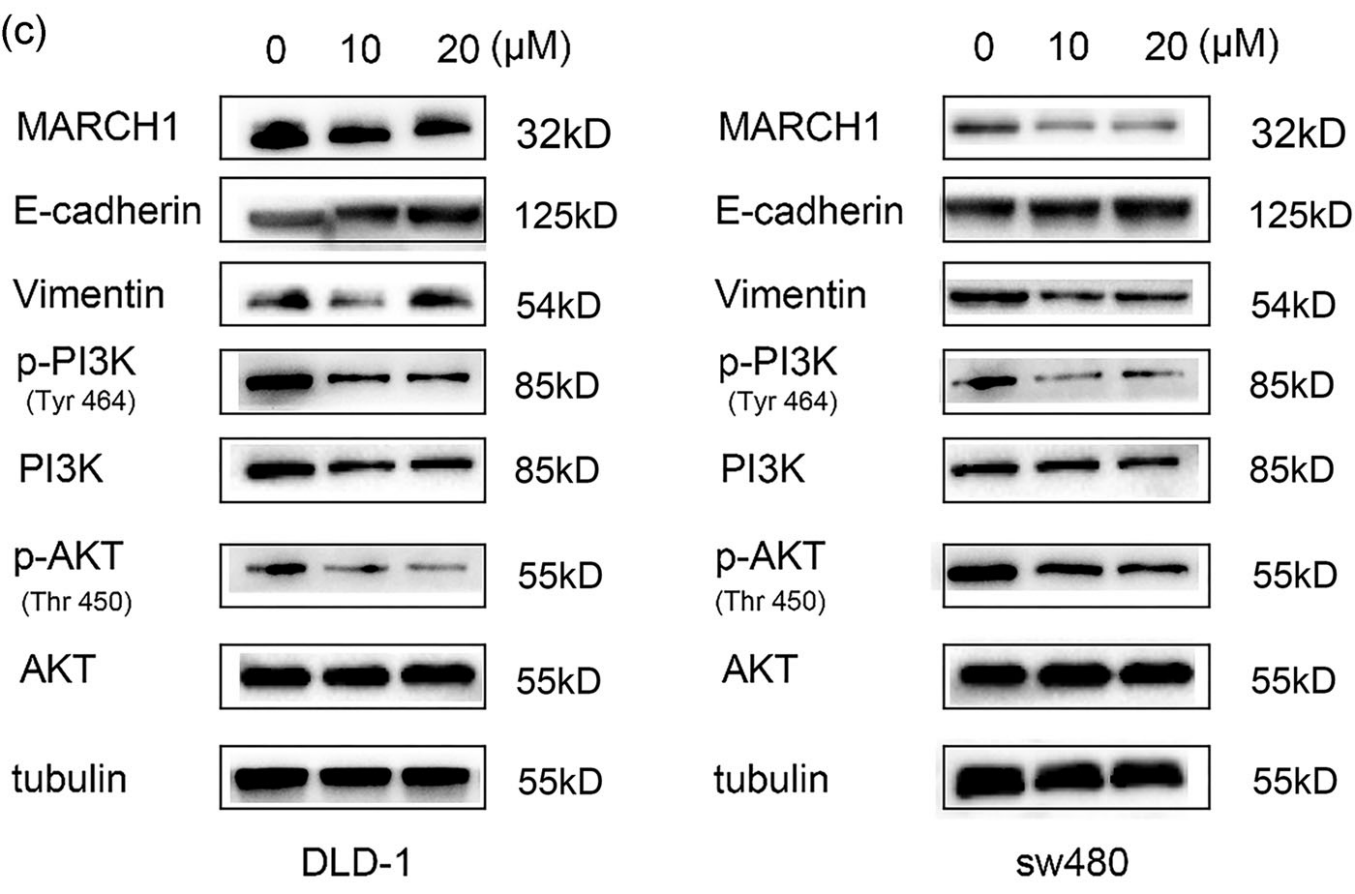

## 4 DISCUSSION

CRC is the leading cause of the sharp increase in cancer morbidity and mortality in the world. The early detection and appropriate treatments have improved patients’ survival in recent decades (Meester et al., 2019). Of note, metastasis is one of the most important events that lead to CRC progression and poor prognosis. Therefore, the investigation of the mechanisms behind metastasis, looking for biomarkers of CRC, and the development of better therapeutics targeting specific molecules are strategies of great significance for the prevention and treatment of CRC.

MARCH1 functions as an E3 ubiquitin ligase and plays an important role in the immune system. According to the literature, Foxp3^+^ T regulatory cells (Tregs) suppress dendritic cells (DCs) and antigen presentation via the production of interleukin 10 (IL‐10), promoting the elevation of MARCH1 mRNA levels (Chattopadhyay & Shevach, 2013). Further, another study confirmed that the immunosuppressive effect of IL‐10 on antigen presentation is mediated through induced expression of MARCH1 (Galbas et al., 2012). Of note, MARCH1 can suppress DC maturation, decreasing the expression of costimulatory molecules and MHC class II (MHC‐II) by ubiquitination of CD86 and MHC‐II β‐chains, respectively (Wilson et al., 2018). Therefore, MARCH1‐mediated ubiquitination of MHC‐II molecules has a regulatory effect on the immune responses. Consequently, the expression of MARCH family molecules, with probable oncogenic functions in tumor progression, usually leads to poor outcomes in cancer. MARCH1 has been studied in ovarian cancer and liver cancer. Meng et al. have found that MARCH1 promotes malignant behaviors in ovarian cancer via the upregulation and formation of a positive feedback loop with the NF‐κB pathway, and the simultaneous upregulation of the Wnt/β‐catenin pathway (Meng et al., 2016). Xie et al. reported that MARCH1 regulates the PI3K‐AKT‐β‐catenin pathway in liver cancer and promotes its development (Xie et al., 2019). Curiously, and in contrast, MARCH1 has been shown to suppress bladder cancer growth (Su et al., 2019). In our study, we found that the expression of MARCH1 (mRNA and protein) is upregulated in CRC tumor tissues and CRC cell lines, compared with that in nontumor tissues and an epithelial intestinal cell line, respectively. We further confirmed that knocking down MARCH1 does not affect cell proliferation and apoptosis, which is inconsistent with the reported role of MARCH1 in liver cancer. This indicates that MARCH1 probably plays different roles in different tumors. However, MARCH1 can significantly affect the migration and invasion of CRC cell lines. There is a lack of research on MARCH1 in gastrointestinal tumors. Of note, our experimental results only incriminated MARCH1 in terms of functional phenotypes in CRC, and could not explain the specific molecular mechanisms behind MARCH1‐derived promotion of CRC.

EMT is essentially a biological process in which epithelial cells acquire an interstitial morphology after stimulation by external signals (Nieto et al., 2016). After the tumor cells undergo EMT, their morphology and protein content change, their intercellular adhesion ability is weakened, and their migration ability is enhanced (Pastushenko & Blanpain, 2019). Therefore, EMT is an important contributor to tumor invasion and metastasis. The low expression of E‐cadherin may enhance the invasion and metastasis of CRC. Vimentin is an important marker of mesenchymal cells and is closely related to CRC cell migration, invasion, and EMT. Our results showed that MARCH1 is significantly upregulated in colon cancer, but the mechanism of MARCH1 in CRC metastasis is not clear. This said, in this study, knockdown of MARCH1 upregulated E‐cadherin and downregulated the expression levels of Vimentin, indicating that the downregulation of MARCH1 inhibits EMT. This phenomenon provides a basis for MARCH1 to participate in the migration and invasion of CRC.

Recently, many signaling pathways have been found to be activated in tumorigenesis and cancer development. The PI3K/AKT signaling cascade is an important canonical signaling pathway in CRC (Bahrami et al., 2018). The abnormal regulation of the PI3K/AKT may influence key tumor oncogenes and suppressive genes, which play a significant role in the regulation of various cell functions, including metabolism, proliferation, and protein synthesis (Porta et al., 2014). Arvindhan Nagarajan et al. (2016) found that MARCH1 is a new inhibitor of insulin receptor signaling (INSR) transduction. MARCH1‐mediated ubiquitination of INSR reduces its cell surface expression levels, mediated by the transcription factor FOXO1. Of note, insulin receptor signaling is related to the family of receptor tyrosine kinases (RTKs; Regad, 2015); RTKs are activated by ligand‐induced dimerization, leading to receptor auto‐phosphorylation, tyrosine activation of RTK substrates, and the downstream activation of RTK targets, including the PI3K/AKT signaling pathway (Vella et al., 2018). Previous studies have shown that the PI3K/AKT pathway is closely related to the regulation of CRC cell invasion and EMT. In fact, there are reports in the literature on the relationship between the PI3K/AKT pathway and EMT CRC cell lines (DLD‐1 and sw480; Duan et al., 2018; Liang, 2020; Tsukamoto et al., 2013; Xiao et al., 2020). Therefore, in this study, we used the DLD‐1 and sw480 cell lines to evaluate the effects of MARCH1 on the PI3K/AKT pathway. Western blot analysis results showed that the expression of p‐PI3K and pAKT proteins decreased after MARCH1 knockdown, suggesting that MARCH1 can indeed inhibit the activation of the PI3K/AKT pathway. Therefore, we speculate that the inhibition of the PI3K/AKT signaling pathway secondary to the downregulation of MARCH1 helps to inhibit the migration and invasion of CRC cells and EMT. However, our research has certain limitations. For instance, whether MARCH1 directly regulates the PI3K/AKT signaling pathway via INSR receptor signaling needs to be further explored.

5‐FU is the main drug used to treat CRC. However, in recent years, more and more patients develop resistance to 5‐FU(Zhang et al., 2008). Therefore, due to resistance, the clinical efficacy of 5‐FU is limited; finding new 5‐FU target molecules was never as necessary as it is today. In this study, we show that 5‐FU notably inhibits the proliferation, migration, and invasion of CRC cells. Simultaneously, 5‐FU promotes cell apoptosis. Considering the influence of cell death on the results of wound healing assay and transwell assay, we further verified the 5‐FU treatment on E‐cadherin and Vimentin through western blot analysis assay. The results indicated that 5‐FU inhibited EMT at the molecular level, which revealed that 5‐FU may inhibit migration and invasion by inhibiting EMT. The classic anticancer mechanism of 5‐FU is to block the catalytic process of thymidylate synthase and further interfere with DNA synthesis (Diasio & Harris, 1989). Importantly, in this study, we found that MARCH1 was downregulated after 5‐FU treatment in a dose‐dependent manner, which may indicate a potentially new mechanism of action of 5‐FU treatment in the context of CRC. In addition, we show that 5‐FU can suppress malignant behaviors of CRC cell lines via the downregulation p‐PI3K and p‐AKT. Our results suggest that 5‐FU also exerts an anticancer effect through the inhibition of the MARCH1‐PI3K/AKT‐EMT pathway.

## 5 CONCLUSION

In conclusion, our experiments further solidify the carcinogenic role of MARCH1 in CRC. Importantly, MARCH1 is associated with the malignant biological behavior of migration and invasion in CRC cell lines. MARCH1 could regulate PI3K/AKT pathway and further influence the progression of EMT, which is important in tumor distant metastasis. Furthermore, we also found that 5‐FU was able to suppress the progression of CRC in vitro by downregulating the expression of MARCH1, partially through the inhibition of PI3K/AKT pathway and EMT.

It is worth noting that our study has some limitations. For example, we did not collect enough clinical samples and follow‐up information to confirm the relationship between MARCH1 expression and patients’ prognosis. Our future research will focus on this mechanism. Finally, this study suggests a new target molecule for 5‐FU treatment and lays the foundation for the future development of therapeutic drugs targeting MARCH1.

## Data Availability

The data that support the findings of this study are openly available in [repository name, for example, “figshare”] at [doi], reference number [reference number].
